# Personal Memories and Bodily-Cues Influence Our Sense of Self

**DOI:** 10.3389/fpsyg.2022.855450

**Published:** 2022-06-22

**Authors:** Lucie Bréchet

**Affiliations:** ^1^Department of Basic Neurosciences, University of Geneva, Geneva, Switzerland; ^2^Department of Neurology, Harvard Medical School, Boston, MA, United States

**Keywords:** bodily-self, autobiographical-self, out-of-body experiences, view-points, autonoetic consciousness, fMRI, VR

## Abstract

How do our bodies influence who we are? Recent research in cognitive neuroscience has examined consciousness associated with the self and related multisensory processing of bodily signals, the so-called bodily self-consciousness. A parallel line of research has highlighted the concept of the autobiographical self and the associated autonoetic consciousness, which enables us to mentally travel in time. The subjective re-experiencing of past episodes is described as re-living them from within or outside one’s body. In this brief perspective, I aim to explore the underlying characteristics of self-consciousness and its relation to bodily signals and episodic memory. I will outline some recent behavioral and neuroimaging evidence indicating that bodily cues play a fundamental role in autobiographical memory. Finally, I will discuss these emerging concepts regarding the current understanding of bodily-self, autobiographical-self, their links to self-consciousness, and suggest directions for future research.

## Introduction

The conscious experience of self-related events is perceived as embodied, i.e., our physical body is a central object in the world. We naturally experience and make sense of the world from an inside viewpoint of our physical body (Mach, 1897), yet we can retrieve memories from both within the body (i.e., own eyes/first-person perspective) or outside the body (i.e., observer/third-person perspective) (Nigro and Neisser, 1983; Rubin and Umanath, 2015). An essential aspect of consciousness is its link with a self, which is the subject of conscious experience. Self-consciousness originates at different levels, from a simple (i.e., the minimal level of unconscious experience) to a complex phenomenon (i.e., the core self and the autobiographical self) (Damasio, 1999). The core consciousness occurs when the brain continuously builds a mental representation of the minimal sense of self caused by an interaction with internal or external stimuli. This minimal sense of self is centered on integrating multisensory, interoceptive and exteroceptive bodily processing (e.g., vision, touch, proprioception, vestibular, and visceral signals) in the brain (Blanke et al., 2015; Park and Blanke, 2019). Manipulating the experience of one’s own body experimentally has been challenging. As James (1890), the pioneering psychologist and philosopher, pointed out in the nineteenth century: *“The body is always there.”* However, the recent advances in virtual reality technology enabled the experimental investigation of the global aspects of bodily self-consciousness, including the self-identification, self-location, and first-person perspective (Ehrsson, 2007; Lenggenhager et al., 2007). The bodily-self, which is the most fundamental aspect of self-consciousness, may influence higher cognitive aspects of self-representation, such as the autobiographical-self (Bergouignan et al., 2014; Bréchet et al., 2019, 2020; Tacikowski et al., 2020; Iriye and St. Jacques, 2021).

Conscious experiences, whether occurring from within or outside the body, are not always bound to here and now. What did you eat for breakfast yesterday? Where do you have to go today? When is your meeting tomorrow? The human mind can detach itself from the present moment and mentally travel to the past or imagine the future (Schacter et al., 2012). As Antonio Damasio (1999) defined, *the autobiographical self* represents a mental state derived from retrieving self-relevant memories. No scene captures the human ability to re-experience past events better than the well-known Marcel Proust’s “madeleine moment.” Struggling to remember details of his childhood, Marcel tastes a petite madeleine soaked in lime-blossom tea, “*and suddenly the memory revealed itself. The taste was that of the little piece of madeleine*…*had recalled nothing to my mind before I tasted it. And all from my cup of tea.”* Tulving (1985) associated the subjective possibility to mentally travel in time with autonoetic consciousness, i.e., the sense of self we experience when we subjectively re-experience an event and mentally travel in time. Mental time travel relies on episodic autobiographical memory, which allows humans to mentally detach themselves from a current self-location and consciously identify themselves at another particular place and time (Hassabis et al., 2007; Schacter et al., 2012; Zaman and Russell, 2021).

The relation between the self and episodic autobiographical memory lies at the core of our understanding of consciousness. One’s autobiographical memories and sense of self are closely related. The self, i.e., the subjective feeling that defines us as unique human beings, is a critical component of consciousness (Damasio, 2003). Memory is necessary for everything we do and provides continuity from one moment to another. Memory creates our conscious sense of identity, which we construct based on self-relevant past events. As James (1890) suggested: *“I enter a friend’s room and see on the wall a painting.” At first, I have the strange, wondering consciousness, “surely I have seen that before,” but when or how does not become clear. There only clings to the picture a sort of penumbra of familiarity—when suddenly I exclaim: “I have it, it is a copy of part of one of the Fra Angelico in the Florentine Academy—I recollect it there!”*

## Bodily-Self Influences the Autobiographical-Self

Episodic autobiographical memory studies have been recently conducted using new approaches to control memory encoding and reflect accurate life-like testing outside the laboratory setting. For example, St Jacques and Schacter (2013); Nielson et al. (2015), and Vogel and Schwabe (2016) developed paradigms in which participants encoded real-life events while wearing a camera that automatically took photos. Still, these studies did not integrate the natural occurrence of participants’ physical bodies during memory retrieval. Perceiving one’s own physical body as part of a visual scene, such as seeing one’s hand pointing at a painting during a museum tour or an animal in a zoo, relies on a multisensory integration of proprioceptive, visual, and tactile cues (Blanke et al., 2015). Subjective experiences create a link between episodic autobiographical memory and bodily self-consciousness, which suggests that the multisensory bodily signals may also be relevant to the conscious re-experiencing of self-relevant, past events.

**FIGURE 1:**
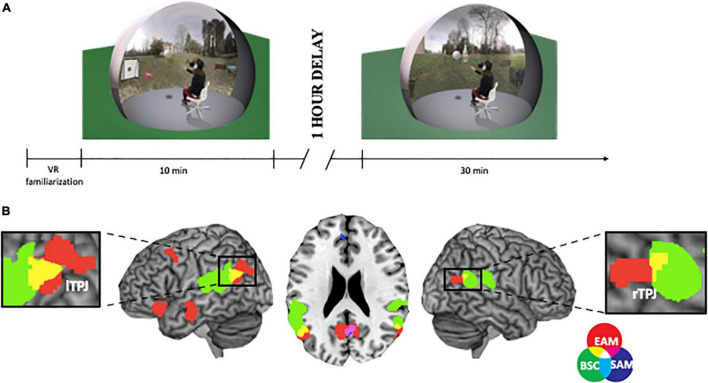
**(A)** Study paradigm. First, participants incidentally learn the context of two different outside scenes (i.e., encoding session; 10 min). Participants were immersed back into the scenes with 1-h delay and were asked to perform a recognition task and subjective confidence ratings for each presented scene (i.e., retrieval session; 30 min). Before the actual study, subjects were seated in a chair, they were asked to put on the HMD and noise-canceling headphones to avoid external disturbances and familiarized themselves with the VR technology (10 min). **(B)** Visualization of the anatomical overlap between BSC fMRI analysis (i.e., self-location and first-person perspective) and EAM ALE analysis bilaterally in the parietal areas Label provided using the MRIcron. Within-cluster FEW-corrected *p* < 0.05 with *p* < 0.001 (uncorrected) as the cluster forming threshold.

Using virtual reality (VR) technology, Bergouignan et al. (2014) tested the encoding of real-life events from within one’s own body/first-person perspective compared to outside one’s own body/third-person perspective. Interestingly, the results showed episodic recollection deficits, specific to the events encoded in the outside body condition, associated with a diminished hippocampal activity (see Table 1). In a follow-up study (Bergouignan et al., 2021), the authors showed that encoding the real-life events from outside one’s own body led to more third-person perspective during the retrieval (see Table 1). Recently, we tested whether the congruent multisensory bodily cues, i.e., the presence or absence of one’s own physical body seen from a first-person perspective, would impact episodic autobiographical memory performance (Figure 1A). We used VR technology to create well-controlled, real life-like scenes into which participants, including their physical bodies, were immersed during the initial stage of encoding and later retrieval (Bréchet et al., 2019). We established that the presence of one’s own physical body during encoding enhanced memory recognition and that this effect was body-specific (see Table 1). In a follow-up study by Gauthier et al. (2020), we demonstrated that seeing one’s own body during encoding impacts the brain mechanisms responsible for episodic autobiographical memory formation by modulating the connectivity between the right hippocampal formation and the neocortical regions involved in the process of multisensory bodily signals and self-consciousness (see Table 1). In line with the previous work, Tacikowski et al. (2020) created an illusion of swapping participant’s own body with a friend’s body and then asked participants to perform personality rating and memory recognition tasks. The authors hypothesized that the perception of one’s own body (bodily-self) would influence beliefs about one’s own personality (conceptual-self) and that a coherent self-representation would lead to normal memory encoding. Indeed, they found that the experience of illusory ownership of a friend’s body changed participants’ beliefs about their own personality and made them more similar to the friend’s personality. Interestingly, they further showed that adjusting to the new bodily-self was beneficial for memory encoding, while incoherence between the bodily-self and conceptual-self leads to memory impairment (see Table 1). The findings from these recent studies point out that memory retrieval is impaired when (i) participants encode events from outside their own bodies, (ii) the body is absent during encoding, and (iii) ownership of one’s own body is reduced during encoding. These novel insights show that a coherent, multisensory representation of one’s own body leads to hippocampal binding mechanisms that are directed to the neocortical areas, which are involved in episodic autobiographical memory (Bergouignan et al., 2014; Gauthier et al., 2020; Iriye and St Jacques, 2020; Roehri et al., 2022).

**TABLE 1:** Studies examining the relationship between bodily-self and autobiographical-self.

Publications	How the aspects of the bodily-self influence the autobiographical self?
Bergouignan et al. (2014)	Out-of-body encoding causes episodic recollection deficits, associated with diminished hippocampal activity
Bergouignan et al. (2021)	Out-of-body encoding leads to more third-person perspective during recollection
Bréchet et al. (2018)	Brain activity related to self-location and 1PP anatomically overlap with episodic ABMs
Bréchet et al. (2019)	Seeing one;s own body during encoding enhances memory recognition
Bréchet et al. (2020)	Body-related integration is important for recall of episodic ABMs and prevents the loss of past events
Gauthier et al. (2020)	Seeing one’s own body during encoding modulates connectivity between hippocampus and neocortical regions
Iriye and St Jacques (2020)	1 PP engages ABM retrieval network (i.e., hippocampus, anterior and posterior midline, frontal and posterior cortices) more strongly than 3 PP
Marcotti and St Jacques (2018)	Shifting visual perspective reduced the accuracy of subsequent memories
Penaud et al. (2022)	Familiarity and self-perspective improve recall and recognition of past events, their spatiotemporal context and sense of remembering
Piolino et al. (2009)	Re-experiencing past events through a feeling of self-awareness and 1 PP is are prone to fading over time
St Jacques et al. (2017)	Shifting visual perspective during ABM retrieval reshapes the characteristics of memories
St Jacques et al. (2018)	Remembering ABMs becomes more like imagination when shifting visual perspective
Tacikowski et al. (2020)	Self-concept can be updated by bodily-self changes; increase in self-coherence facilitates memory encoding

## Bodily-Self and Autobiographical-Self Representations Anatomically Overlap

Until recently, it has been unknown whether bodily self-consciousness and autobiographical memories, either episodic or semantic, involve distinct or similar brain regions. We examined whether experimental manipulation of the self-location and first-person perspective aspects of bodily self-consciousness may anatomically overlap with the activations related to the subjective conscious experience of remembering self-relevant past events (Bréchet et al., 2018). We included results from patients suffering from out-of-body experiences with abnormal self-location and first-person perspective, whose brain damage was localized in the inferior parietal lobule (Ionta et al., 2011). Our systematic meta-analysis of neuroimaging studies revealed an anatomical overlap bilaterally in the angular gyrus, specific to bodily-self and episodic autobiographical-self, while there was no overlap with semantic autobiographical-self (see Figure 1B and Table 1). This finding is supported by the emerging evidence from studies in healthy participants that shows that the lateral parietal cortex is critical for the subjective, conscious experience of retrieving multisensory episodic memories (Bonnici et al., 2016, 2018; Sestieri et al., 2017; Humphreys et al., 2021). Related to this, recent series of papers (St Jacques et al., 2017, 2018; Marcotti and St Jacques, 2018; Iriye and St Jacques, 2020) showed that shifting visual perspective during retrieval shapes accuracy and subjective vividness of memories, which activated the posterior parietal cortex (see Table 1). Emerging evidence from patients with lateral parietal lesions shows that damage to this brain region causes reduced subjective confidence, vividness, and richness when re-experiencing self-relevant memories (Simons et al., 2010; Berryhill, 2012; Hower et al., 2014) as well as egocentric episodic memory deficits (Russell et al., 2019). Recently, Penaud et al. (2022) showed that familiarity and self-perspective (i.e., centered on one’s own interaction with the environment) improved recall of past events, their spatiotemporal context and one’s sense of remembering (see Table 1).

## Bodily-Self Retroactively Strengthens Autobiographical-Self

Many seemingly irrelevant everyday life events may become significant only later (Kensinger, 2015). For example, that stranger who asked for directions becomes more relevant after realizing that your wallet is missing. Recently, two behavioral studies showed how memory for neutral images could be enhanced by future fearful (Dunsmoor et al., 2015) or rewarding (Patil et al., 2017), conceptually related events. More precisely, during the first stage of incidental encoding, the so-called *“pre-conditioning classification task,”* two neutral categories of images portraying animals and tools appeared to be of the same relevance. During a second phase of the incidental encoding, the so-called *“conditioning classification task,”* a prominent event, either fear conditioning or reward motivation, became purposefully associated with one of the two categories (animals or tools). A memory recognition task revealed that participants remembered better neutral images (for example, tools) associated with fear or reward during the conditioning phase. They also remembered better the conceptually related images (tools) from the pre-conditioning phase.

Studies on mental self-projections (Arzy et al., 2008; Dafni-Merom and Arzy, 2020) suggest that the experience of the self in the present moment is also involved with the ability to remember our past or imagine the future. Furthermore, this conscious self-awareness (i.e., a mental state in which the content of one’s consciousness refers to knowledge about oneself, for example, reflecting about one’s personality or identity) is intrinsically connected to the multisensory bodily processes (Blanke et al., 2015; Tacikowski et al., 2020). Therefore, we examined whether the retroactive and selective effect could be (a) triggered by the multisensory bodily signals, such as the presence or absence of one’s own physical body and (b) generalized to naturalistic scenes, such as inside rooms or outdoor scenes, into which the participants would be immersed using VR technology (Bréchet et al., 2020). We showed that the presence of one’s own body can retroactively strengthen memory recognition and that this retroactively enhancing effect became selectively associated with a particular group of items (either from rooms or scenes) (see Table 1).

## Summary and Future Directions

The behavioral and neuroimaging evidence reviewed here suggests that the fundamental aspects of self-consciousness, the bodily-self and autobiographical-self, critically interact and impact each other. We experience the world from an inside viewpoint of a body and from a physical location of a body, which we identify as our own. This sense of ownership that I am the one who currently experiences the world around me is also essential with respect to one’s past and future, therefore playing a significant role in constructing an autobiographical self. However, only a handful of recent studies (see Table 1) examined the relationship between bodily-self and autobiographical-self experimentally and showed that encoding events (i) from outside one’s own body, (ii) when one’s body is absent, (iii) when the sense of ownership of one’s body is reduced leads to impairment of episodic autobiographical memories. On the other hand, these studies also revealed the coherent multisensory bodily-self representations have beneficial, strengthening effects on autobiographical memory and may prevent memory loss.

Understanding the interactions between the core aspects of self-consciousness, bodily-self, and autobiographical-self has important clinical implications. Several studies have found that memory decline in aging is associated with a lack of vivid autobiographical memories and increased retrieval from a third-person perspective (Piolino et al., 2006, 2009). Both normal aging and Alzheimer’s disease in autobiographical memory are highly related to the self (Martinelli and Piolino, 2009; Martinelli et al., 2013). Alzheimer’s disease is a neurodegenerative, progressive disorder that distorts the autobiographical-self, which is tightly connected to the sense of agency (“I am the one who generates experiences”) and ownership (“I am the one who undergoes experiences”) in the world (Arzy and Schacter, 2019). Detecting these early bodily-self signs, which are often overlooked in Alzheimer’s disease, is of importance. One of the challenges for future research will be to reinstate a coherent sense of self to reduce autobiographical memory impairment.

Several open questions arise from these recent studies and their results. How can the neuroscientific methods ensure that people selectively remember what’s significant to them in real life rather than laboratory-based settings? How can we ensure that only some memories are enhanced while others are diminished? Would those mentioned above, experimental studies tested in healthy young participants, modify the bodily-self and autobiographical-self of Alzheimer’s patients in the same way? Is this the first step toward *“a memory pacemaker”*? More work is needed toward the long-term vision of restoring memory function. And perhaps more philosophically: What is more important: bodily-self or autobiographical-self? Quoting the words of Wiesel (2012): *“Illness may diminish me, but it will not destroy me. The body is not eternal, but the idea of the soul is. The brain will be buried, but memory will survive it.”*

## Author Contributions

The author confirms being the sole contributor of this work and has approved it for publication.

## Conflict of Interest

The author declares that the research was conducted in the absence of any commercial or financial relationships that could be construed as a potential conflict of interest.

## Publisher’s Note

All claims expressed in this article are solely those of the authors and do not necessarily represent those of their affiliated organizations, or those of the publisher, the editors and the reviewers. Any product that may be evaluated in this article, or claim that may be made by its manufacturer, is not guaranteed or endorsed by the publisher.
